# Revisiting differential control of sympathetic outflow by the rostral ventrolateral medulla

**DOI:** 10.3389/fphys.2022.1099513

**Published:** 2023-01-17

**Authors:** Soumya S. Kulkarni, Nicholas A. Mischel, Patrick J. Mueller

**Affiliations:** Department of Physiology, Wayne State University School of Medicine, Detroit, MI, United States

**Keywords:** RVLM, Glutamate, GABA, blood pressure regulation, lateralization

## Abstract

The rostral ventrolateral medulla (RVLM) is an important brain region involved in both resting and reflex regulation of the sympathetic nervous system. Anatomical evidence suggests that as a bilateral structure, each RVLM innervates sympathetic preganglionic neurons on both sides of the spinal cord. However, the functional importance of ipsilateral *versus* contralateral projections from the RVLM is lacking. Similarly, during hypotension, the RVLM is believed to rely primarily on withdrawal of tonic gamma aminobutyric acid (GABA) inhibition to increase sympathetic outflow but whether GABA withdrawal mediates increased activity of functionally different sympathetic nerves is unknown. We sought to test the hypothesis that activation of the ipsilateral *versus* contralateral RVLM produces differential increases in splanchnic *versus* adrenal sympathetic nerve activities, as representative examples of functionally different sympathetic nerves. We also tested whether GABA withdrawal is responsible for hypotension-induced increases in splanchnic and adrenal sympathetic nerve activity. To test our hypothesis, we measured splanchnic and adrenal sympathetic nerve activity simultaneously in Inactin-anesthetized, male Sprague-Dawley rats during ipsilateral or contralateral glutamatergic activation of the RVLM. We also produced hypotension (sodium nitroprusside, i.v.) before and after bilateral blockade of GABA_A_ receptors in the RVLM (bicuculline, 5 mM 90 nL). Glutamate (100 mM, 30 nL) injected into the ipsilateral or contralateral RVLM produced equivalent increases in splanchnic sympathetic nerve activity, but increased adrenal sympathetic nerve activity by more than double with ipsilateral injections *versus* contralateral injections (*p* < 0.05; *n* = 6). In response to hypotension, increases in adrenal sympathetic nerve activity were similar after bicuculline (*p* > 0.05), but splanchnic sympathetic nerve activity responses were eliminated (*p* < 0.05; *n* = 5). These results provide the first functional evidence that the RVLM has predominantly ipsilateral innervation of adrenal nerves. In addition, baroreflex-mediated increases in splanchnic but not adrenal sympathetic nerve activity are mediated by GABA_A_ receptors in the RVLM. Our studies provide a deeper understanding of neural control of sympathetic regulation and insight towards novel treatments for cardiovascular disease involving sympathetic nervous system dysregulation.

## 1 Introduction

The sympathetic nervous system is taught classically to mediate the “fight-or-flight” response by increasing the activity of sympathetic nerves that directly innervate the heart, blood vessels, and adrenal glands (Boron and Boulpaep, 2017). Indeed, global activation of the sympathetic nervous system increases heart rate, cardiac contractility, vasoconstriction and release of epinephrine (McAllen and Dampney, 1990; Guyenet, 2006). Sympathetic nerves originate in the spinal cord and are innervated directly by a brain region known as the rostral ventrolateral medulla (RVLM), named for its anatomical location in the brainstem. The RVLM contains neurons that are critical for moment-to-moment regulation of resting blood pressure *via* tonic sympathetic drive to most if not all organs (Guyenet, 2006; Schreihofer and Sved, 2011). Furthermore, it is well-established that the RVLM regulates sympathetic nerve activity (SNA) differentially to each organ, based on the physiological demands of the body (McAllen et al., 1995; Morrison and Gebber, 2001; Mueller et al., 2011). Despite the importance of the RVLM in physiological and pathophysiological states, the functional anatomy and neurotransmitter mechanisms that allow the RVLM to regulate these differential outputs remain to be fully elucidated.

As alluded to above, a subpopulation of sympathoexcitatory neurons in the RVLM maintain resting blood pressure *via* a tonic level of activity (Guyenet, 2006; Schreihofer and Sved, 2011). However, most if not all RVLM neurons appear to be under significant modulation by the inhibitory neurotransmitter transmitter, gamma aminobutyric acid (GABA), in part by input from the arterial baroreceptor reflex to maintain beat-by-beat regulation of blood pressure (Schreihofer and Guyenet, 2002; Guyenet and Stornetta, 2004). Increases and decreases in GABAergic inhibition of the RVLM provide bidirectional modulation of excitatory drive to most sympathetic targets (Schreihofer and Guyenet, 2002; Guyenet and Stornetta, 2004). In the context of the capacity of the RVLM to differentially regulate functionally different outputs, specific anatomical and neurotransmitter mechanisms that allow such precise control of blood pressure are not entirely clear.

Anatomically, the RVLM exists as a bilateral structure, with each RVLM projecting to sympathetic preganglionic neurons on both sides of the spinal cord in varying degrees (Pyner and Coote, 1998; Moon et al., 2002). For example, *via* retrograde tracing techniques, Moon and others (2002) observed that a single RVLM innervated sympathetic preganglionic neurons in the superior cervical ganglia, including those on both left and right sides of the spinal cord. In contrast to the superior cervical ganglia, Moon et al. also reported that an individual RVLM provided primarily ipsilateral innervation of sympathetic preganglionic neurons projecting to the adrenal medulla (Moon et al., 2002). Despite the importance of other anatomical studies (Wesselingh et al., 1989; Jansen et al., 1995; Pyner and Coote, 1998), the relative influence of ipsilateral *versus* contralateral projections of the RVLM on functionally relevant changes in sympathetic outflow to the diverse targets are not fully known.

In addition to differences in degree of lateralization, distinct subpopulations of neurons in the RVLM may be responsible for regulating sympathetic nerve activity to various end organs. For example, as reviewed by McAllen and colleagues (1995), several studies have mapped the RVLM of the cat and shown subpopulations of neurons which control muscle vasoconstrictor, cutaneous vasoconstrictor, and renal sympathetic outflow (McAllen et al., 1995). The hypothesis that subpopulations of neurons in the RVLM are functionally segregated has been explored across several species (McAllen et al., 1995; Ootsuka and Terui, 1997; Sved et al., 2001; Mueller et al., 2011). However, whether subpopulations of neurons involved in regulating functionally diverse sympathetic targets can be separated further functionally on the basis of anatomical or neurotransmitter-based mechanisms remains to be explored more fully.

Glutamate and GABA are the primary fast neurotransmitters in the RVLM, playing unique and interacting roles in modulating sympathetic nerve activity *via* the presence of respective receptors on bulbospinal neurons (Mueller et al., 2020; Fyk-Kolodziej et al., 2021). On one hand, glutamate mediates most sympathoexcitatory reflexes (Guyenet, 2006; Schreihofer and Sved, 2011). However, withdrawal of tonic GABAergic inhibition of sympathoexcitatory neurons in the RVLM has long been considered the primary mechanism for baroreflex-mediated increases in sympathetic nerve activity (Sun and Guyenet, 1985; Dampney, 1994; Mueller, 2007). While both glutamate and GABA play significant roles in the RVLM, the more precise mechanisms by which they influence sympathetic nerve activity to functionally diverse organs are not yet entirely clear.

In the present study, we hypothesized that given the diverse physiological roles of sympathetic nerves, the RVLM employs both unique and overlapping mechanisms to regulate functionally distinct targets. More specifically, we tested whether bulbospinal projection patterns or tonic inhibitory mechanisms mediate differential regulation of splanchnic SNA (as an example of sympathetic nerve activity involved in direct vasoconstriction) and adrenal SNA (as an example of sympathetic nerve activity with more complex regulation of blood pressure as well as blood glucose regulation) at the level of the RVLM. The goals of the present study were to: 1) compare sympathoexcitatory effects of contralateral *versus* ipsilateral activation of the RVLM on splanchnic SNA *versus* adrenal SNA, and 2) determine the role of tonic GABAergic inhibition of the RVLM in mediating hypotension-induced increases in splanchnic SNA *versus* adrenal SNA. We explored these aims by activating the RVLM either directly with glutamate or indirectly by inducing hypotension and recording from the adrenal and splanchnic sympathetic nerves simultaneously in the same set of animals.

## 2 Materials and methods

All experiments presented in this paper received prior approval from the Wayne State University Institutional Animal Care and Use Committee of Wayne State University (Animal Welfare Assurance Number A3310-01). Procedures involving animals were also carried out in accordance with the Guide for the Care and Use of Laboratory Animals, published by the National Institutes of Health. All animals were euthanized *via* injections of Fatal Plus (0.2 mL, i.v. Vortech Pharmaceuticals, Dearborn, MI), which produced asystole as observed from the recorded pulse pressure. Secondary assurances of death included thoracotomy and decapitation (for brain removal). Similar to our previous studies (Mueller et al., 2020; Fyk-Kolodziej et al., 2021), our experimental design was directed to minimize the number of animals used to test statistical significance for primary outcomes. Experiments were performed on male Sprague Dawley rats (Harlan, Indianapolis, IN; 300–400 g; *n* = 6) at 16 weeks of age.

All procedures were consistent with previous experiments from our laboratory and have been described in detail elsewhere (Mueller et al., 2011; Dombrowski and Mueller, 2017). Briefly, animals were group-housed under a standard 12-h light-dark cycle under temperature-controlled conditions and food and water were provided *ad libitum*. All animals were overdosed as described above and the brain was removed and fixed in a solution of 4% phosphate buffered formalin.

### 2.1 Sympathetic nerve activity recordings

The procedures for sympathetic nerve recordings have been documented in detail in previous work by our laboratory (Mischel and Mueller, 2011; Mueller et al., 2011; Subramanian and Mueller, 2016; Dombrowski and Mueller, 2017). In brief, rats were anesthetized initially with isoflurane (5% induction, 2% maintenance; Henry Schein, Melville, NY) to levels that resulted in an absence of withdrawal to firm toe pinch. Catheters were inserted into the left femoral artery to monitor arterial pressure and into the femoral vein for infusion of anesthesia and drugs. A tracheostomy was performed and animals were ventilated artificially with settings based on each animal’s body weight, which were entered into the ventilator (Harvard Inspira, Harvard Instruments, Holliston, MA). The left splanchnic and left adrenal nerves were exposed *via* a retroperitoneal approach. The adrenal nerves were identified by visually tracing their entry directly to the adrenal gland. The splanchnic nerves were identified by tracing the adrenal nerve/vascular bundle proximally to the point where the postganglionic portion of the splanchnic nerves course caudally and medially. Electrodes were implanted around both nerves and embedded in gel (Kwik-Sil, World Precision Instruments, Sarasota, FL).

Anesthesia was transitioned over to the long-acting anesthetic Inactin by an intravenous infusion (100 mg/kg iv; Sigma Aldrich, St. Louis, MO), followed by supplemental doses (5–10 mg iv) to maintain a lack of response to corneal stimulation or to firm toe pinch. A water-circulated heating pad was used to maintain rectal temperature between 37°C and 38°C (model K20; Baxter, Valencia, CA). Blood gases were monitored and if adjustments were required, changes in rate (up to 80 breaths per minute) or volume were made to maintain pCO_2_ between 35–40 mmHg. The vagus nerve remained intact for all experiments.

Raw adrenal and splanchnic sympathetic nerve activities were monitored on an oscilloscope and audio monitor. Ganglionic blockers (30 mg/kg hexamethonium and 1 mg/kg atropine methyl bromide, both iv) were injected at the end of the experiment to determine background noise in the splanchnic nerve recordings and post-mortem recordings were used to determine background noise in adrenal SNA. Values obtained after administration of the ganglionic blockers or post-mortem were subtracted from the nerve recordings obtained during the experiment to obtain absolute voltages for SNA. Before producing sympathetic activation with either microinjections of glutamate into the RVLM or systemic hypotension with sodium nitroprusside, all nerve activity was considered at physiological baseline and changes in sympathetic nerve activity were calculated as absolute or percent change from baseline.

### 2.2 RVLM microinjections

Animals were placed into a stereotaxic device (Kopf) to position them consistently, and to properly orient the head for microinjections into the RVLM. To expose the brainstem, the skin was incised and the muscle overlying the foramen magnum was retracted. We removed a small portion of the occipital bone, and then incised and reflected the dura mater to expose the surface of the brainstem. In all experiments, we used single-barrel glass micropipettes inserted at a 90° angle (perpendicular) to the brainstem surface. Microinjections were delivered *via* a pressure microinjection system (Pneumatic Picopump PV820; World Precision Instruments, New Haven, CT). The appropriate volume of drug was delivered during each microinjection by observing the meniscus level of the micropipette using a 150x compound microscope with a calibrated reticle. At the end of experiments, microinjections of Chicago Sky Blue dye (2%, 30 nL) were performed to localize injections sites, similar to our previous studies (Mischel and Mueller, 2011; Subramanian and Mueller, 2016; Dombrowski and Mueller, 2017).

### 2.3 Direct glutamatergic activation of the RVLM

Coordinates previously confirmed by our laboratory were used to begin localizing the left and right RVLM (1.0 mm rostral and 1.8 mm lateral to calamus scriptorius, and 3.2 mm ventral to the dorsal surface of the medulla) (Dombrowski and Mueller, 2017). Small adjustments of 100–200 μm were made until correct placement of the micropipette produced an increase of at least 10 mmHg in blood pressure using microinjections of 100 mM glutamate (30 nL/injection).

Animals received glutamate (100 mM, 30 nL injections unilaterally into the ipsilateral (left) or the contralateral (right) RVLM, order randomized. Five minutes was allowed between injections to minimize tachyphylaxis. In a previous study (Mueller, 2007), we have already demonstrated that injections of larger volumes of drug vehicle (90 nL, artificial cerebrospinal fluid) produced only minor fluctuations in arterial pressure (<5 mmHg).

### 2.4 Hypotension-induced sympathoexcitation

Ramp infusions of sodium nitroprusside (200 μg/mL) were administered *via* the femoral venous catheter until decreases in mean arterial pressure (MAP) of 40–60 mmHg were produced (Mischel and Mueller, 2011). We began at 2.5 μg·kg^−1^·min^−1^ and increased the infusion rate in graded steps to produce a gradual reduction in MAP over 1–2 min. The graded constant infusions were performed to enable sympathetic nerve activity to reach a steady-state. The graded constant infusions were delivered at doses of sodium nitroprusside ranging from 2.5 to 80 μg·kg^−1^·min^−1^. The total volume of sodium nitroprusside administered during decreases in MAP was between 300 and 450 µL.

Subsequently, we performed bilateral GABA_A_ receptor blockade by administering either bicuculline (5 mM, 90 nL, *n* = 5) or gabazine (2 mM, 90 nL, *n* = 4). The dosage for bicuculline was determined in previous studies by our laboratory (Mueller, 2007; Mueller and Mischel, 2012) to be sufficient to block activation of endogenous GABA receptors at baseline and in response to decreases in blood pressure. Following bilateral GABA_A_ receptor blockade, the sodium nitroprusside protocol was repeated to produce equivalent decreases in arterial pressure.

### 2.5 Data analysis

Sympathetic nerve activity (SNA) recordings, arterial pressure (AP), and heart rate (HR) were collected and analyzed with PowerLab (PowerLab; ADInstruments, Colorado Springs, CO) and Chart software (version 6; ADInstruments). Raw, multiunit splanchnic SNA and adrenal SNA were recorded at 10 kHz and filtered using a band pass of 30 Hz to 3 kHz. We rectified and integrated the raw SNA signals using a 28 ms time constant. The processed SNA was then filtered at 1 Hz to obtain an average level of splanchnic SNA and adrenal SNA. We calculated background noise following ganglionic blockade for splanchnic SNA and post-mortem recordings were used to determine background noise for adrenal SNA. We then subtracted background noise from the average level of splanchnic SNA and adrenal SNA. Arterial pressure and heart rate are expressed as absolute changes (Δ mmHg and Δ beats/min) and changes in splanchnic SNA and adrenal SNA are expressed as a percentage of baseline and as absolute voltage (mV·s) (Mischel and Mueller, 2011; Matus et al., 2021).

Statistical analysis was performed using Sigma Stat 3.5 (Systat Software, Chicago, IL). Absolute levels of MAP (mmHg), splanchnic SNA (mV·s) and adrenal SNA (mV·s) both before and after glutamate microinjections in the ipsilateral or contralateral RVLM were analyzed by a two-way repeated measures ANOVA, with baseline *versus* peak values as one main effect (repeated measures) and ipsilateral *versus* contralateral as the second main effect (also with repeated measures). Absolute values for MAP, splanchnic SNA and adrenal SNA both before and after hypotension produced by sodium nitroprusside under control conditions and in the presence of GABA_A_ receptor blockade in the RVLM with bicuculline were analyzed similarly with a two repeated measures ANOVA. Preliminary experiments with another GABA_A_ receptor antagonist, gabazine, were not powered statistically as a primary outcome and results of the two-way repeated measures ANOVA are noted in the Results section. MAP, splanchnic SNA and adrenal SNA values expressed as percent of baseline in each set of experiments were also analyzed by two-way repeated measures ANOVA to account for variances in resting voltages between and across nerves. When a significant interaction occurred between main effects (e.g. baseline *versus* peak and ipsilateral *versus* contralateral microinjection), a *post hoc* Holm-Sidak was used to test for individual differences.

To examine absolute and percent changes in MAP, splanchnic SNA and adrenal SNA, paired Student’s *t*-tests were performed on ipsilateral *versus* contralateral microinjections, effects of bicuculline and effects of gabazine, again the latter not statistically powered as a primary outcome. We set the threshold for significance at *p* < 0.05 and listed exact *p* values above 0.001 and values below 0.001 as *p* < 0.001, per results generated by SigmaStat.

### 2.6 Drugs

Isoflurane was obtained from Henry Schein (Melville, NY). Inactin, L-glutamate, bicuculline methiodide, sodium nitroprusside, hexamethonium, and atropine were obtained from Sigma Chemical (St. Louis, MO). L-glutamate, bicuculline, and gabazine were dissolved in artificial cerebrospinal fluid at a pH of 7.3–7.5.

## 3 Results

### 3.1 Activation of the ipsilateral *versus* the contralateral RVLM produces greater increases in adrenal but equivalent increases in splanchnic sympathetic nerve activity and blood pressure

**FIGURE 1:**
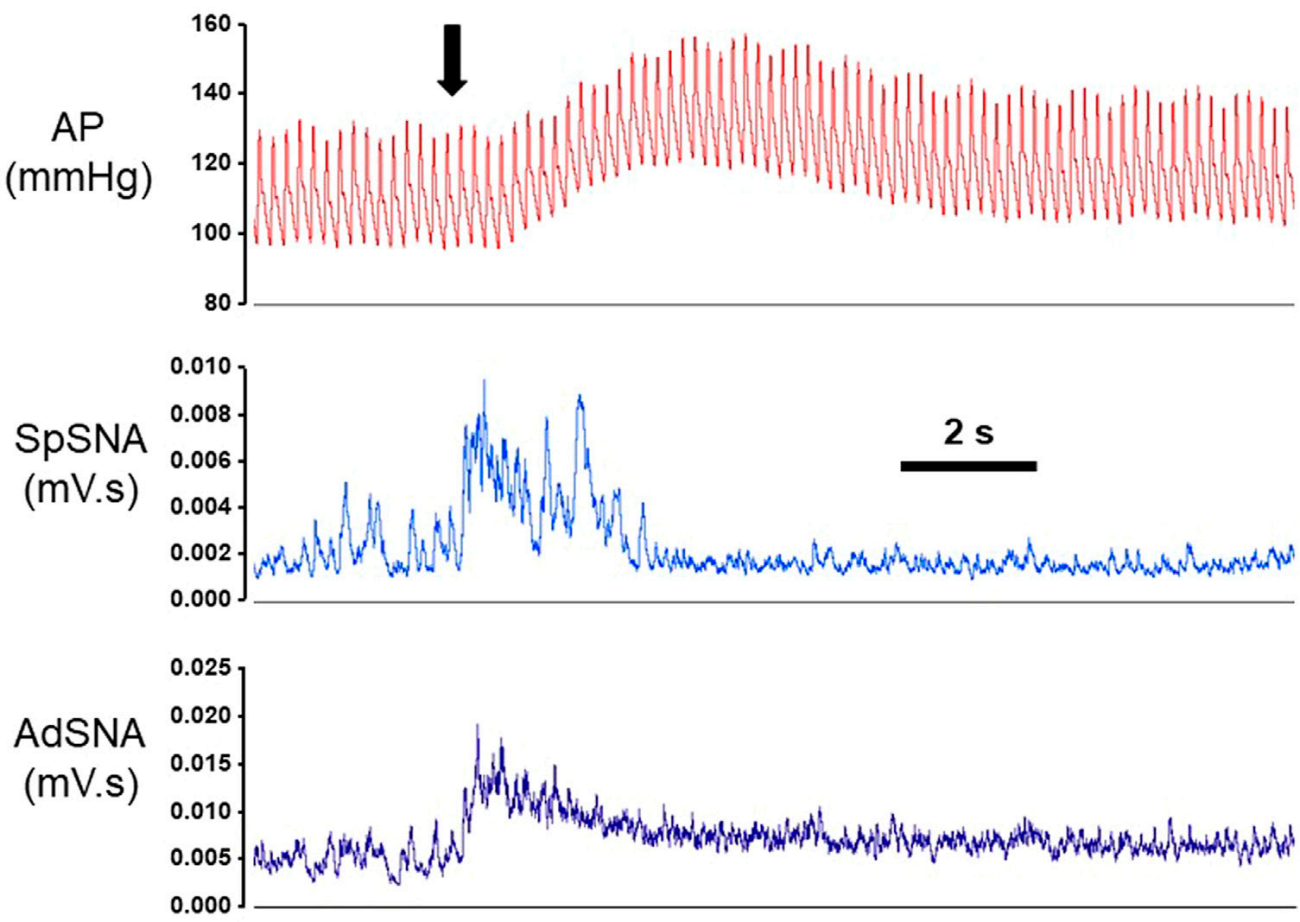
Examples of beat-by-beat blood pressure (AP), splanchnic sympathetic nerve (SpSNA) and adrenal sympathetic nerve (AdSNA) recordings in one animal during a single microinjection of glutamate into the rostral ventrolateral medulla (RVLM). Following microinjection of glutamate (100 mM, 30 nL; denoted by arrow) in the RVLM, SpSNA, and AdSNA increased almost immediately and preceded subsequent increases in AP. Abbreviations: mV·s; millivolt-seconds of integrated SNA. Scale bar represents 2 s.

Contralateral and ipsilateral injections of glutamate (100 mM, 30 nL) were used to activate the left and right RVLMs, respectively, while measuring arterial pressure (AP), mean arterial pressure (MAP), heart rate (HR), splanchnic SNA, and adrenal SNA. An example of a glutamate microinjection into the ipsilateral RVLM of an individual animal is shown in Figure 1. As expected from previous studies, glutamate microinjections into the RVLM, produced near immediate increases in splanchnic SNA and adrenal SNA, while increases in blood pressure occur shortly thereafter and predictably.

**FIGURE 2:**
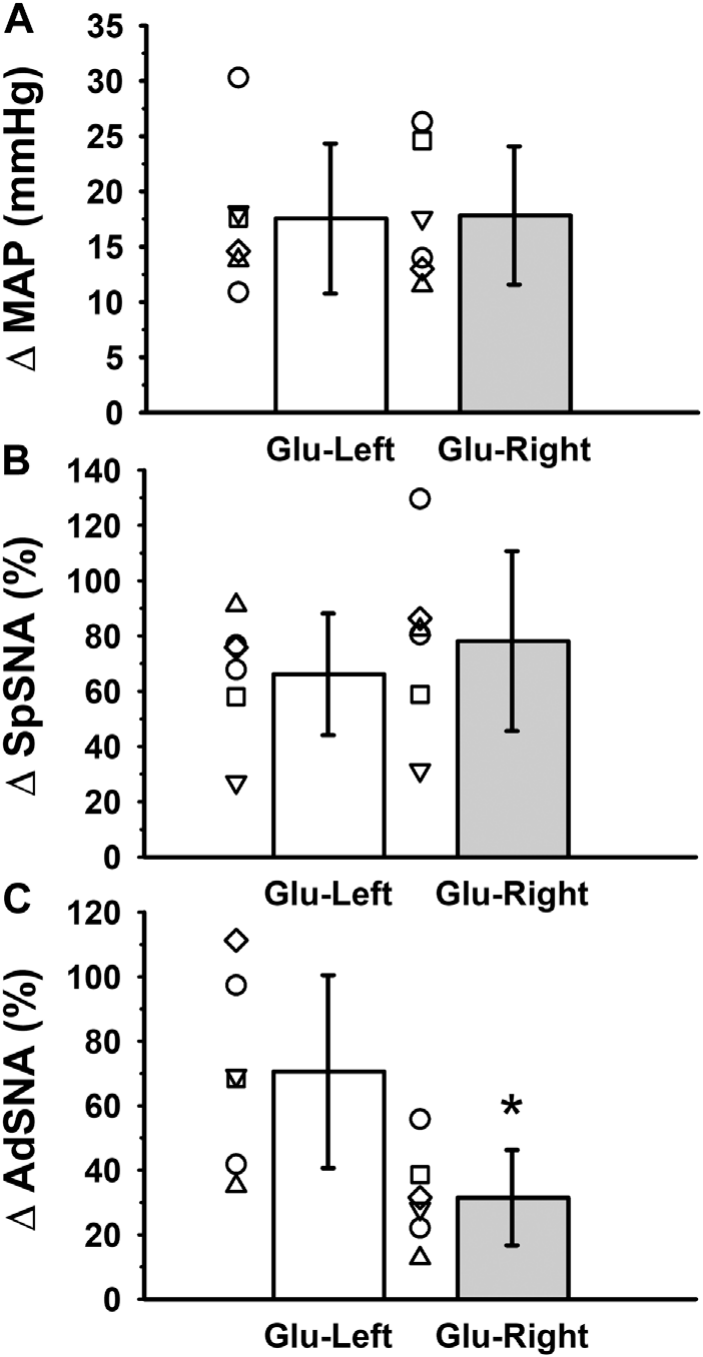
Group data from six experiments in which glutamate was microinjected into the left or right RVLM during simultaneous measurements of **(A)** mean arterial pressure (MAP) and **(B)** left SpSNA and **(C)** left AdSNA. Glutamate injections into either the right or left RVLM produced significant increases in MAP from baseline (Table 1; *p* < 0.001; main effect of glutamate), which when calculated as absolute change were not significantly different between left and right injections (Figure 2A; *p* = 0.942; paired *t*-test). Similarly, increases in SpSNA calculated as percent change were also not different between ipsilateral and contralateral injections (Figure 2B; *p* = 0.470; paired *t*-test), suggesting equivalent capacities of each RVLM to increase arterial blood pressure and SpSNA when activated by glutamatergic mechanisms. In contrast, increases in AdSNA calculated as percent change were significantly greater after ipsilateral compared to contralateral injections (Figure 2C; *, *p* = 0.017; paired *t*-test), suggesting a greater capacity of the ipsilateral RVLM to increase sympathetic outflow to the adrenal gland *via* glutamatergic mechanisms. Abbreviations as defined in Figure 1 legend.

Similar to individual responses shown in Figure 1, glutamate injections into either the right or left RVLM in six rats produced significant increases in MAP from baseline (Table 1; *p* < 0.001; main effect of glutamate), which were similar whether performed on the side ipsilateral (left) or contralateral (right) relative to the side of SNA recordings (*p* = 0.882; main effect of side). There was no significant interaction between baseline/post-glutamate conditions and sidedness of injections (*p* = 0.871), which precluded further *post hoc* analysis of absolute levels of MAP. Changes in absolute MAP were not significantly different between ipsilateral *versus* contralateral injections (Figure 2A; *p* = 0.942; paired *t*-test), suggesting equal capacities of both RVLMs to increase arterial blood pressure when activated by glutamatergic mechanisms.

**TABLE 1:** Absolute values before and after microinjections of glutamate in the left or right RVLM, or after intravenous injections of sodium nitroprusside before and after bilateral microinjections of bicuculline into the RVLM.

		Baseline MAP	Peak MAP	Baseline SpSNA	Peak SpSNA	Baseline AdSNA	Peak AdSNA
Glutamate	(n)	(mmHg)	(mmHg)	(µV**·**s)	(µV**·**s)	(µV**·**s)	(µV**·**s)
Left RVLM	6	119 ± 13	137 ± 16*	3.2 ± 2.0	5.5 ± 3.5^#^	3.9 ± 1.6	6.3 ± 2.0*^$^
Right RVLM	6	119 ± 12	137 ± 15*	3.1 ± 1.8	5.9 ± 4.0^#^	3.9 ± 1.6	5.0 ± 1.8*

SNP + bicuculline		Baseline MAP	Peak MAP	Baseline SpSNA	Peak SpSNA	Baseline AdSNA	Peak AdSNA
	(n)	(mmHg)	(mmHg)	(µV**·**s)	(µV**·**s)	(µV**·**s)	(µV**·**s)
SNP	5	114 ± 8	49 ± 6*	2.7 ± 1.3	3.5 ± 2.1	5.3 ± 2.9	6.1 ± 3.4
Bic	5	114 ± 12	151 ± 15*	2.5 ± 1.1	3.4 ± 1.4	5.1 ± 2.7	7.5 ± 5.4
SNP Post Bic	5	148 ± 12$	62 ± 13*^	3.2 ± 1.4	3.3 ± 1.4	6.5 ± 3.7	7.9 ± 4.7

n, number of observations; MAP, mean arterial pressure; mmHg = millimeters of mercury; SpSNA, splanchnic sympathetic nerve activity; AdSNA, adrenal sympathetic nerve activity; μV·s = integrated μV seconds; SNP, sodium nitroprusside; Bic = bicuculline; data are mean + STD. *, *p* < 0.001 (main effect for MAP, compared to baseline for left or right glutamate in RVLM, or *post hoc* Holm-Sidak for AdSNA, compared to baseline for left or right glutamate in RVLM, or *post hoc* Holm-Sidak for MAP, response to SNP, compared to baseline for both conditions and for MAP compared to baseline after Bic.; #, *p* = 0.022 compared to baseline; $, *p* < 0.001 compared to AdSNA, levels after Right RVLM, injection of glutamate (*post hoc* Holm-Sidak for AdSNA, response to glutamate) or compared to baseline MAP, prior to SNP; ^, *p* = 0.025 (*post hoc* Holm-Sidak for MAP, levels after Bic + SNP, compared to after SNP, alone).

Similarly, absolute levels of splanchnic SNA (mV·s) were significantly increased compared to baseline when glutamate was injected into the RVLM whether ipsilateral or contralateral to the splanchnic SNA recording (Table 1; *p* < 0.022; main effect of glutamate). Overall absolute levels of splanchnic SNA were not significantly different between ipsilateral and contralateral experiments (*p* = 0.664; main effect of side). There was also no significant interaction between baseline/post-glutamate conditions and sidedness of microinjections (*p* = 0.207), which precluded further *post hoc* analysis. Increases in splanchnic SNA calculated as percent change were also not different between ipsilateral and contralateral microinjections (Figure 2B; *p* = 0.470), suggesting equivalent capacities of both RVLMs to increase splanchnic SNA when activated by glutamatergic mechanisms.

In contrast, although glutamate injections into either the ipsilateral or contralateral RVLM produced significant increases in absolute adrenal SNA (mV·s) from baseline (Table 1; *p* < 0.001; main effect of glutamate), absolute levels of adrenal SNA following glutamate microinjections were dependent on the side injected (*p* = 0.001; main effect of side). A significant interaction between baseline/post-glutamate conditions and sidedness of injections (*p* < 0.001) justified further *post hoc* testing, and revealed that peak levels of absolute adrenal SNA were significantly higher after ipsilateral compared to contralateral microinjections of glutamate (Table 1; *p* < 0.001; Holm-Sidak), despite similar baseline absolute levels of adrenal SNA (Table 1; *p* = 0.828). Increases in adrenal SNA calculated as percent change were significantly greater after ipsilateral compared to contralateral microinjections (Figure 2C; *p* = 0.017; paired *t*-test), suggesting a greater capacity of the ipsilateral RVLM to increase sympathetic outflow to the adrenal gland *via* glutamatergic mechanisms.

### 3.2 Hypotension-induced changes in splanchnic but not adrenal sympathetic nerve activity require GABA_A_ receptors in the RVLM

**FIGURE 3:**
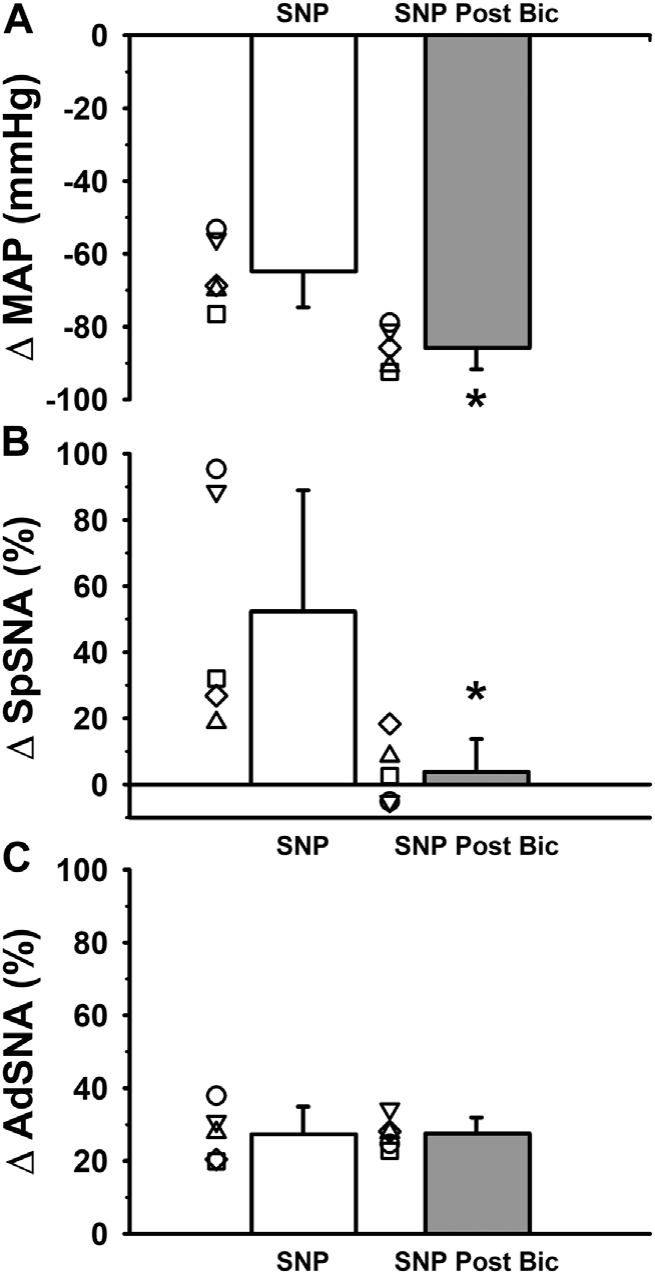
Group data from five experiments in which responses to intravenous administration of sodium nitroprusside were recorded during simultaneous measurements of **(A)** mean arterial pressure (MAP) and **(B)** left SpSNA and **(C)** AdSNA. Averaged data from experiments in which sympathoexcitatory responses to intravenous administration of sodium nitroprusside (SNP) were compared under control conditions and following bilateral microinjection of the GABA_A_ receptor antagonist, bicuculline (2 mM, 90 nL per side) into the RVLM. Intravenous SNP produced significant decreases in MAP from baseline (Table 1; *p* < 0.001; main effect of glutamate). When calculated as absolute changes in MAP, the decrease in blood pressure in response to SNP was significantly greater in the presence of bicuculline compared to control conditions (Figure 3A; *, *p* = 0.004; paired *t*-test), suggesting the same dose of SNP produced enhanced depressor responses, likely due to a reduced capacity to reflexively increase sympathetic activity in at least some vascular beds (see below). SNP-induced changes in SpSNA calculated as percent change were significantly reversed when measured in the presence of bicuculline when compared to control conditions (Figure 3B; *, *p* = 0.021; paired *t*-test). In contrast, SNP-induced increases in AdSNA calculated as percent change were not significantly different measured in the presence of bicuculline and compared to control conditions (Figure 3C, *p* = 0.969; paired *t*-test). Abbreviations as defined in Figure 1 legend.

Administration of sodium nitroprusside resulted in absolute levels of MAP that were significantly lower than baseline whether performed under control or following bilateral microinjections of bicuculline (Table 1; *p* < 0.001; main effect of sodium nitroprusside). Absolute levels of MAP also appeared to be significantly different between control or bicuculline conditions (*p* = 0.004; main effect of bicuculline) and a significant interaction between the main effects of sodium nitroprusside and bicuculline (*p* < 0.001) justified additional *post hoc* analyses. Consistent with the main effect of sodium nitroprusside, sodium nitroprusside produced significantly lower MAP compared to baseline when sodium nitroprusside responses were examined individually under control conditions or following microinjections of bicuculline (Table 1; *p* < 0.001 for both; Holm-Sidak). As expected, baseline blood pressures were significantly higher after giving bilateral bicuculline into the RVLM (*p* < 0.001) and likely contributed to significantly higher levels of MAP following sodium nitroprusside administration in the presence of bicuculline (Table 1; *p* = 0.025). When calculated as absolute changes in MAP, the decrease in blood pressure in response to sodium nitroprusside was significantly greater in the presence of bicuculline compared to control conditions (Figure 3A; *p* = 0.004), suggesting the same dose of sodium nitroprusside produced enhanced depressor responses, likely due to a reduced capacity to reflexively increase sympathetic activity in at least some vascular beds (see below).

Baseline measures of absolute splanchnic SNA appeared to increase following sodium nitroprusside-induced and hypotension-induced depressor responses (Table 1; *p* < 0.061; main effect of sodium nitroprusside). In addition, absolute levels of splanchnic SNA were not significantly different between control conditions and following bicuculline microinjections into the RVLM (*p* = 0.801; main effect of side). The lack of a significant interaction between the main effects of sodium nitroprusside administration and bicuculline microinjection (*p* = 0.232) precluded further *post hoc* testing. Sodium nitroprusside-induced and hypotension-induced changes in splanchnic SNA calculated as percent change were significantly blocked when splanchnic SNA responses were measured in the presence of bicuculline compared to control conditions (Figure 3B; *p* = 0.021; paired *t*-test), suggesting GABA_A_ receptors in the RVLM are necessary for hypotension-induced changes in splanchnic SNA.

Baseline levels of absolute adrenal SNA increased significantly following sodium nitroprusside-induced decreases in MAP (Table 1; *p* < 0.038; main effect of sodium nitroprusside). The main effect of bicuculline microinjections on absolute levels of adrenal SNA did not reach statistical significance (*p* = 0.055), and the lack of a significant interaction between the main effects of sodium nitroprusside administration and bicuculline microinjection (*p* = 0.125), precluded any further *post hoc* testing. Interestingly, sodium nitroprusside-induced increases in adrenal SNA calculated as percent change were not significantly different in the presence of bicuculline compared to control conditions (Figure 3C; *p* = 0.969; paired *t*-test), suggesting that GABA_A_ receptors in the RVLM are not required for hypotension-induced increases in adrenal SNA.

We also tested the requirement of GABA_A_ receptors during sympathoexcitation produced by hypotension using microinjections of the structurally distinct GABA_A_ receptor antagonist, gabazine, in a subset of animals (*n* = 4). Although not powered statistically as a primary outcome of our study, we observed relatively similar effects between gabazine and bicuculline. For example, gabazine significantly elevated baseline MAP compared to control conditions (*p* = 0.034) and produced a greater fall in MAP in response to sodium nitroprusside compared to control conditions (*p* = 0.003). Similar to bicuculline, gabazine was also ineffective at altering the adrenal SNA responses to sodium nitroprusside compared to control conditions (*p* = 0.453).

### 3.3 Histology

**FIGURE 4:**
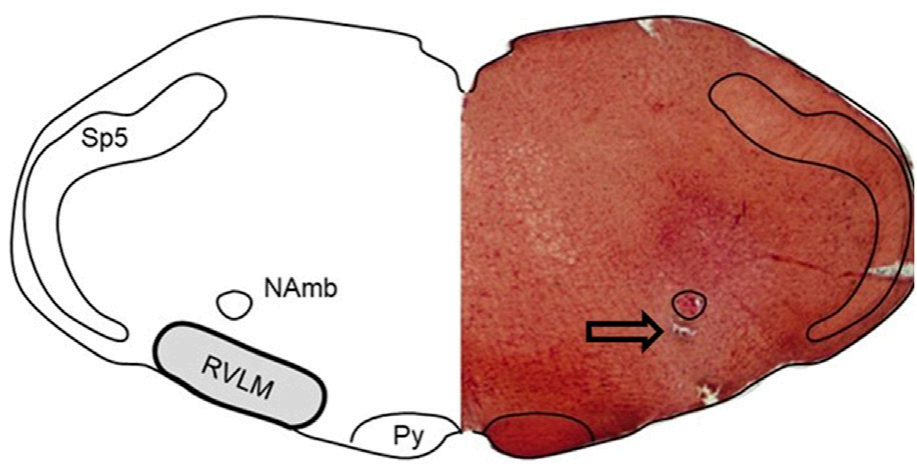
Histological verification of injection site. Representative photomicrograph of a neutral red-stained hemi-section (right) juxtaposed with a diagram outlining the anatomical landmarks designating the region of the RVLM (left). The RVLM is well-defined anatomically in the literature by others and us (Dampney, 1994; Guyenet, 2006; Mischel & Mueller, 2011; Schreihofer & Sved, 2011; Subramanian & Mueller, 2016) as being located within 500 μm of the caudal pole of the facial motor nucleus; the area ventral to the compact portion of the nucleus ambiguus (NA); lateral to the pyramidal tracts (py); and medially to the spinotrigeminal nucleus (Sp5). The anatomical representation (left) was created by tracing the neutral-red stained hemi-section, and structures were identified with the aid of a standard rat atlas (Paxinos and Watson, 2007). Arrow represents location of the pipette tip just ventral to the compact portion of the NA.

In addition to functional verification of the RVLM with glutamate microinjections, microinjection sites were verified histologically by injections of Chicago Sky Blue dye (2%, 30 nL), similar to our previous studies (Dombrowski and Mueller, 2017) (Mischel and Mueller, 2011; Subramanian and Mueller, 2016). Figure 4 contains a representative photomicrograph of a neutral red-stained hemi-section (right) juxtaposed with a diagram outlining the anatomical landmarks designating the region of the RVLM (left). The tip of the microinjection pipette was located either just above or within the region just ventral to the compact portion of the nucleus ambiguus, lateral to the pyramidal tracts and medial to the spinal trigeminal nucleus.

## 4 Discussion

The experiments presented in this paper help further our understanding of the control of sympathetic nervous system activity by the RVLM, with implications in both physiological and pathophysiological maintenance of blood pressure homeostasis. Our key findings are as follows: 1) We found that activation of the RVLM that is ipsilateral to sympathetic nerves being recorded resulted in significantly larger increases in adrenal SNA as compared to activation of the contralateral RVLM. 2) In contrast, we found that during the very same ipsilateral or contralateral injections, activation of the RVLM produced equivalent increases in simultaneously-recorded splanchnic SNA. 3) We also found that blockade of GABA_A_ receptors prevented hypotension-induced changes in splanchnic SNA. 4) However, the increase in simultaneously-recorded adrenal SNA during hypotension was unaffected by GABA_A_ receptor blockade. Our results suggest that the RVLM may differentially regulate adrenal *versus* splanchnic SNA *via* the degree of contralateral projections to respective sympathetic preganglionic neurons in the spinal cord. Our findings also suggest that the RVLM increases adrenal SNA in response to hypotension *via* mechanisms other than GABAergic disinhibition. Finally, similar to other sympathetic nerves, responses in splanchnic nerve activity in response to hypotension appear to be mediated by withdrawal of GABAergic inhibition. Collectively, our results are supported by previous studies and continue to refine the originally-held understanding that the sympathetic nervous system exhibits an “all-or-none” response. Instead we and others suggest that the RVLM tailors regulation of sympathetic nerve activity more finely to address the physiological circumstances at hand *via* anatomical and neurotransmitter-based mechanisms (McAllen et al., 1995; Morrison and Gebber, 2001; Sved et al., 2001).

Greater increases in adrenal SNA produced by ipsilateral *versus* contralateral activation of the RVLM provide important functional relevance toward previous anatomical studies. For example, using trans-synaptic viral tracing techniques, Wesselingh and colleagues (1987) and later Jansen and colleagues (1995) demonstrated innervation of the adrenal gland by the RVLM and several other brain regions. Subsequently, utilizing combined anterograde and retrograde tracing techniques, Pyner and Coote (1998) and later Moon and colleagues (2002) also described a population of RVLM neurons projecting to the sympathetic preganglionic neurons that innervate the adrenal medulla (Pyner and Coote, 1998). Moon and colleagues (2002), appear to be the first to quantify the degree of lateralized connections between the adrenal gland and the RVLM using single-sided injections of anterograde and retrograde tracers into the RVLM and adrenal medulla, respectively. The authors observed that the RVLM provide predominantly ipsilateral innervation of sympathetic preganglionic neurons, which innervate the adrenal medulla (Moon et al., 2002). Although the authors found anatomical evidence of primarily lateralized connections, evidence supporting functional relevance of differences in lateralization was still lacking (see below).

Interestingly, in the same study by Moon and colleagues (2002), microinjections of glutamate into the ipsilateral or contralateral RVLM appeared to produce similar but variable increases in adrenal SNA in a small cohort of animals. In the present study, we were able to demonstrate a significant, functional lateralization in the RVLM’s regulation of adrenal *versus* splanchnic SNA using a larger sample size and in Inactin (*versus* urethane) anesthetize rats. To our knowledge, these results are the first to suggest a functionally important difference in the predominantly ipsilateral innervation of the adrenal medulla by pathways originating in the RVLM. It is reasonable to hypothesize that the predominantly ipsilateral innervation of the adrenal medulla could allow the RVLM to preferentially activate one adrenal gland more readily under certain circumstances such as damage or injury occurring on one side of the brainstem or spinal cord.

Equally important are findings demonstrating similar capacities of both ipsilateral and contralateral RVLMs to increase splanchnic SNA. Our studies are supported by previous studies examining sympathetic output to other sympathetic targets. For example, glutamatergic activation of the ipsilateral or contralateral RVLM produced equivalent increases in lumbar SNA in anesthetized rats (Mueller, 2007). Furthermore, anatomical studies have demonstrated similar numbers of retrogradely labeled neurons in the ipsilateral and contralateral RVLM following tracer injections at T4 and T10 levels of the spinal cord (Gowen et al., 2012). Although the retrograde tracer injections at different levels of the spinal cord were not targeted to organ-specific SNAs, they provide corroborative data towards the hypothesis that an individual RVLM has the functional capacity to activate both ipsilateral and contralateral SNA pathways. Our functional studies add to this hypothesis by suggesting that each RVLM activates ipsilateral and contralateral SNA pathways equivalently in some but not all SNAs, specifically when SNA responses are compared simultaneously during microinjections of glutamate. Further studies utilizing both anatomical and physiological techniques are certainly needed to better understand how contralateral projections from the RVLM function under physiological and pathophysiological circumstances, but certain inferences can be made from existing studies in the literature (see below).

As a bilateral structure, both the left and right RVLM serve to maintain resting blood pressure in anesthetized animals (Guyenet, 2006; Schreihofer and Sved, 2011). In fact, when isolated experimentally, a single intact RVLM appears to have a capacity to maintain resting blood pressure alone (Horiuchi and Dampney, 1998; Dombrowski and Mueller, 2017). For example, inhibition of one RVLM in anesthetized animals can decrease resting blood pressure; however, bilateral inhibition of the RVLM typically decreases resting blood pressure to levels similar to those observed in spinally-transected animals (Ito and Sved, 1996; Horiuchi and Dampney, 1998; Huber and Schreihofer, 2011). Interestingly, blockade of one RVLM also enhances sympathoinhibitory responses to acute inhibition of the remaining, intact RVLM during microinjections of GABA (Dombrowski and Mueller, 2017). Furthermore, enhanced sympathoinhibitory response to GABA occurred in sedentary but not physically active rats (Dombrowski and Mueller, 2017). Collectively, these findings not only reinforce the importance of the RVLM in blood pressure regulation under physiological circumstances, but suggest important considerations of the remaining, intact RVLM under pathophysiological circumstances where unilateral damage to the brainstem has occurred.

Related to the above, is also important to acknowledge the dependence of the RVLM on the arterial baroreflex in maintenance of resting blood pressure, which is mediated primarily by GABA-mediated inhibition and disinhibition (Guyenet, 2006; Schreihofer and Sved, 2011). The reliance on the arterial baroreflex to maintain blood pressure by the RVLM is most evident in studies in which inhibition of one RVLM in baroreceptor denervated animals causes resting blood pressure to fall to spinal levels (Horiuchi and Dampney, 1998). In addition, since baroreflex buffering of blood pressure is dependent on GABA receptors in the RVLM (Sun and Guyenet, 1985; Dampney, 1994; Mueller, 2007), it is also not surprising that blocking GABA_A_ receptors in the RVLM enhances subsequent pressor and sympathoexcitatory responses to glutamate microinjections (Mischel and Mueller, 2011). Intriguingly, enhanced pressor responses to glutamate after GABA_A_ receptor blockade occurs only in sedentary rats; whereas pressor responses in physically active rats was unchanged by GABA_A_ receptor blockade (Mueller and Mischel, 2012). Thus, examination of the neurotransmitter mechanisms by which each RVLM regulates diverse sympathetic outputs deserves additional attention under both physiological and pathophysiological circumstances.

In the present study, only changes in splanchnic SNA were abolished by bilateral blockade of GABA_A_ receptors in the RVLM. In previous studies, hypotension-induced increases in lumbar sympathetic nerve activity were also abolished by blockade of GABA_A_ receptors in the RVLM (Sun and Guyenet, 1985; Dampney, 1994; Mueller, 2007). In other studies, blockade of GABA_A_ receptors in the RVLM resulted in loss of cardiac-related (presumably baroreceptor driven) rhythmicity in renal sympathetic nerve activity (Tagawa et al., 1999). Collectively, these studies suggest GABAergic mediated inhibition of RVLM neurons involved in control of splanchnic sympathetic nerve activity is primarily driven at least in part by arterial baroreceptor pathways and mediated by GABA_A_ receptors in the RVLM.

Similar to our previous study (Mueller et al., 2011), we confirmed in the present study that adrenal SNA increased in response to hypotension. However, unlike splanchnic SNA, increases in adrenal SNA in response to hypotension were unaffected by bilateral blockade of GABA_A_ receptors in the RVLM with bicuculline. Therefore, we suggest that the RVLM may utilize different neurotransmitter systems to regulate adrenal SNA under baseline conditions compared to conditions of baroreceptor unloading. One possibility is that adrenal SNA increases in response to glutamate release in the RVLM following decreases in blood pressure. Previously, (Mayorov and Head, 2001), demonstrated that baroreflex-mediated increases in renal sympathetic nerve activity in conscious rabbits were attenuated by RVLM microinjections of kynurenic acid, an ionotropic glutamate antagonist. Similarly, other studies have shown the increases in adrenal SNA produced by hyperinsulinemia (Bardgett et al., 2010) or peripheral administration of 2-deoxyglucose (Korim et al., 2016) are also reduced by microinjection of a glutamate receptor antagonist in the RVLM. We speculate that glutamate may drive additional adrenal sympathoexcitation during decreases in blood pressure in the presence or absence of GABAergic inhibition. However, further studies are necessary to confirm our hypothesis.

There are important technical considerations in studying the physiology of differential control of sympathetic outflow. As noted above, unilateral activation of the RVLM with microinjections, can help suggest important mechanisms in regulation of sympathetic outflow but are limited by compensatory mechanisms involving the contralateral RVLM (Dombrowski and Mueller, 2017). In addition, since there are both anatomical and functional studies suggesting that one RVLM projects to the contralateral RVLM (McMullan and Pilowsky, 2012; Turner et al., 2013), we need to interpret our results and those of others cautiously.

In the present study we are unable to distinguish influence of axon collaterals projecting to different SNAs (Barman and Gebber, 1985); albeit presumed to be in the minority (Jansen et al., 1995; Farmer et al., 2019). Also, similar to our previous studies, we left the vagus nerves intact to preserve as much of the integrative physiological response as possible. We recorded only from left splanchnic and left adrenal sympathetic nerves, due to technical feasibility of bilateral recordings. Our conclusions also pertain only to male rats, yet sex-related differences in control of SNA is an important and timely topic. To this end, we have published a recent study related to sex-differences in control of splanchnic SNA based on age, sexual maturity and sedentary *versus* physically active conditions (Matus et al., 2021). Given the results of the current study, a series of future experiments involving multi-nerve recordings during activation of the RVLM in female rats is certainly warranted. Finally, in each of the conditions mentioned above, it is important to include caveats and limitations for every study and to interpret results cautiously.

There are also certain considerations in studying differential control of SNA using pharmacological tools. One of the strengths of our study is that we used glutamate as the well-known endogenous excitatory neurotransmitter of RVLM neurons (Sved et al., 2001; Schreihofer and Sved, 2011). We purposefully used glutamate to activate receptors already shown to be present in the RVLM *via* a variety of techniques (Mueller et al., 2020; Fyk-Kolodziej et al., 2021). Similarly, previous studies from our laboratory and others confirm the existence of GABA_A_ receptors in the RVLM using anatomical, biochemical and functional techniques (Mueller, 2007; Mueller and Mischel, 2012; Dombrowski and Mueller, 2017; Pawar et al., 2017), including on bulbospinal neurons (Mueller et al., 2020). Lastly, we were also careful to use two different GABA_A_ receptor antagonists in separate protocols to block tonic inhibition of the RVLM by endogenous GABA. We based our use and doses of these drugs on previous studies that verified have the selectivity of bicuculline and gabazine (Barman and Gebber, 2007). In hindsight, injecting GABA into the RVLM would have made for intriguing, yet challenging interpretations due the possibility of differential receptor expression across neurons *versus* global inhibition of neurons with varying levels of tonic activity. We recommend future studies keep these challenges in mind when using pharmacological approaches in studying the regulation of blood pressure and SNA by the RVLM.

## 5 Summary

Summarizing the findings of the present study, we suggest that the RVLM regulates adrenal SNA and splanchnic SNA differentially *via* at least two distinct mechanisms, which are not necessarily accounted for as overtly or at all in previous studies: 1) Anatomically, the RVLM influences blood pressure control *via* the number and functional influence of ipsilateral *versus* contralateral projections to the spinal cord, and 2) RVLM neurons express and utilize distinct neurotransmitter receptors in its regulation of different sympathetic outflows. To what extent these mechanisms relate to other sympathetic targets and their more overt involvement in normal and disease states has yet to be determined.

**FIGURE 5:**
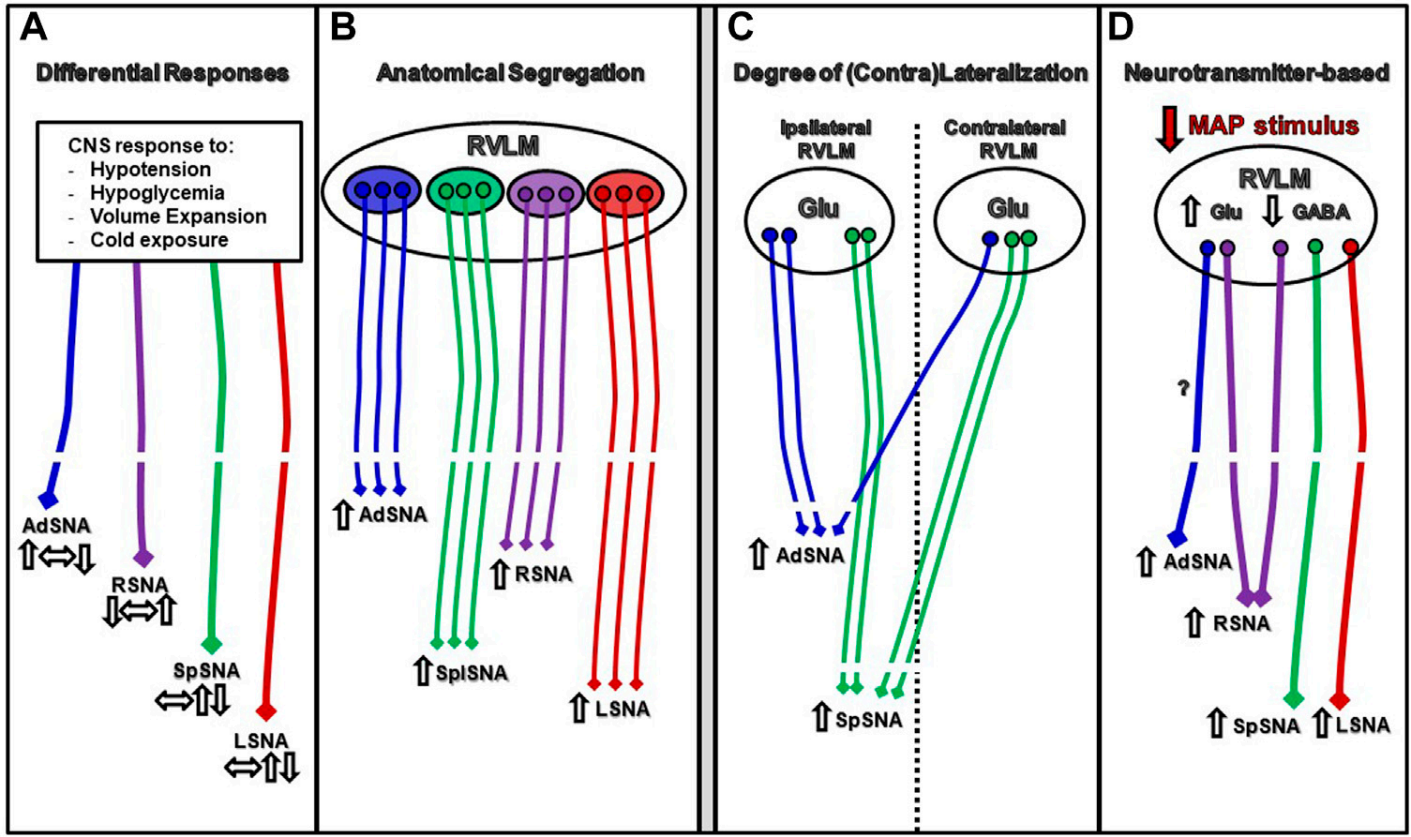
Schematic representations of some of the proposed mechanisms by which the RVLM regulates sympathetic outflow in a differential manner. Panel **(A)** Diverse physiological stimuli (i.e., hypoglycemia, volume expansion, etc. ref) trigger mechanisms within the CNS to elicit differential responses in sympathetic nerves of varying magnitude and direction. Panel **(B)** Individual neurons in RVLM subregions may exert control over individual nerves and be parcellated anatomically (McAllen et al., 1995; Sved et al., 2001). Panel **(C)** The bilateral structures of the RVLM have the capacity to control ipsilateral and bilateral nerve activity; however, there appears to be preferentially control of ipsilateral nerve activity in some cases (AdSNA) (Figure 2). Panel **(D)** Autonomic reflexes like the baroreflex may utilize different neurotransmitter systems to differentially regulate different vascular beds under conditions of hypotension (Figure 3; Mayorov and Head, 2001).

More broadly, understanding of how regulation of sympathetic outflow is achieved by the RVLM has progressed well beyond the original idea of an all-or-none concept, originated *via* the flight-or-fight response. Instead, the “management” of sympathetic outflow by the RVLM appears to occur *via* several sophisticated, yet complementary mechanisms. Figure 5 depicts a simplistic representation of only some of the proposed mechanisms by which the RVLM regulates sympathetic outflow in a differential manner. For simplicity sake, only a few possibilities are represented here with the intent to stimulate discussion of refinement and yet-to-be discovered possibilities by future experimentation. Similarly, the reader is referred to other work reporting evidence of individual RVLM neurons having more broad control over multiple sympathetic targets *via* axon collaterals that terminate in different parts of the spinal cord (Barman and Gebber, 1985); presumed to be in the minority of bulbospinal neurons (Jansen et al., 1995; Gowen et al., 2012; Farmer et al., 2019).

Investigating the underlying mechanisms behind differential regulation of SNA will not only lead to a clearer understanding of normal physiology, but may help guide therapeutic approaches in the treatment of pathophysiological circumstances. For example, in hypertension and other disease states associated with increased sympathetic nerve activity, it is evident that specific sympathetic pathways can either contribute to the disease state; compensate or remain permissive (Osborn and Kuroki, 2012). More directly, a randomized control trial in drug-resistant hypertension patients showed that some forms of neurogenic hypertension can be well managed by selective denervation of the renal sympathetic nerves (Symplicity et al., 2010). In contrast, rat models of salt-sensitive neurogenic hypertension did not benefit from renal denervation - in these models, the splanchnic sympathetic nerves were a better therapeutic target (Osborn and Kuroki, 2012). However, stimulation of the postganglionic splanchnic nerves has been shown to produce both mesenteric vasoconstriction and hindquarter vasodilation (Sato et al., 2006). Before selective denervation and manipulation of sympathetic nerves can become a mainstream therapeutic strategy, it will be critical to elucidate these nuanced mechanisms of differential sympathetic nerve control. Similarly, the recent advances in spinal cord stimulation therapies in the management hypotensive crisis (Squair et al., 2021), suggests a need for a more intimate knowledge of differences in projection patterns and the plasticity of sympathetic nerves regulating different vascular beds in developing these novel therapies. Thus, there is a fundamental need to better understand differential control of sympathetic nerve regulation in the context of achieving safe and more effective therapies in the future.

## Data Availability

The raw data supporting the conclusions of this article will be made available by the authors, without undue reservation.
